# “It’s called overamping”: experiences of overdose among people who use methamphetamine

**DOI:** 10.1186/s12954-022-00588-7

**Published:** 2022-01-16

**Authors:** Robert W. Harding, Katherine T. Wagner, Phillip Fiuty, Krysti P. Smith, Kimberly Page, Karla D. Wagner

**Affiliations:** 1 School of Public Health, University of Nevada, Reno, 1664 N. Virginia St. MC 0274, Reno, NV 89509 USA; 2 University of New Mexico Health Sciences Center, Albuquerque, NM USA; 3 The Mountain Center Harm Reduction Center, Santa Fe, NM USA

**Keywords:** Methamphetamine, Overdose, Polysubstance use, Qualitative methods

## Abstract

**Background:**

The USA is experiencing increases in methamphetamine use and methamphetamine-related or attributed deaths. In the current study, we explore qualitative narratives of methamphetamine overdose and strategies used by people who use drugs to reduce the undesirable effects associated with methamphetamine use.

**Methods:**

We conducted 21 qualitative interviews with people over the age of 18 who reported using methamphetamine in the previous 3 months in Nevada and New Mexico. Interviews were recorded, transcribed, and analyzed using qualitative thematic analysis.

**Results:**

Respondents described a constellation of psychological and physical symptoms that they characterized as “overamping,” experienced on a continuum from less to more severe. Reports of acute, fatal methamphetamine overdose were rare. Few reported seeking medical attention for undesirable effects (usually related to psychological effects). General self-care strategies such as sleeping and staying hydrated were discussed.

**Conclusions:**

When asked directly, our respondents claimed that acute, fatal methamphetamine overdose is rare or even impossible. However, they described a number of undesirable symptoms associated with overconsumption of methamphetamine and had few clinical or harm reduction strategies at their disposal. Addressing this current wave of drug-related deaths will require attention to the multiple factors that structure experiences of methamphetamine “overdose,” and a collaborative effort with PWUDs to devise effective harm reduction and treatment strategies.

## Background

Amphetamine type stimulants account for a significant share of illicit drug use globally [1–5]. Among amphetamine type stimulants, the most widely and commonly used is methamphetamine [5]. In the early 2000s, the highest prevalence of methamphetamine use was in Asian Regions [2]. Since then, methamphetamine use has been expanding in other regions of the globe [3, 4]. The UN Office on Drugs and Crime has been noting increasing use of amphetamine type stimulants, particularly methamphetamine, for over a decade [2]. In 2019, an estimated 27 million persons worldwide used methamphetamine, with the highest prevalence occurring in North America [3].

In fact, the USA experienced a 198% increase in use of amphetamine type stimulants between 2010 and 2019, driven primarily by methamphetamine [6]. Following earlier trends in opioid-related morbidity and mortality, where initial increases were most dramatic in smaller rural communities, rural and non-metro counties have also seen the largest increases in methamphetamine use per capita [7–9]. In the USA, rural and non-metro counties typically have far fewer substance use disorder treatment, harm reduction, and healthcare services, making adequate response to drug-related morbidity and mortality more difficult. Despite the higher rates of use in rural counties compared to urban, most methamphetamine research in the USA has been conducted with urban sexual minorities in the context of HIV prevention research [10–13] and has not sufficiently explored the phenomenon of methamphetamine use among other populations of users.

Use of methamphetamine is associated with multiple morbidities, including: psychiatric symptoms (hallucinations, delusions, anxiety, and depression) [14, 15], physical concerns (hypertension, increased risk of intracranial hemorrhagic stroke, cardiomyopathy, renal failure, ischemic heart disease), and increased risk of suicide and injury [15–20]. In recent years, US surveillance reports have identified increases in methamphetamine-related hospital and substance use disorder admissions. For example, from 2010 to 2017 in Washington state in the Western USA, the age-adjusted rate for methamphetamine-involved hospitalizations increased from 6.3 to 8.5/100,000 [21]. Nationally, hospital admissions for heart failure involving methamphetamine increased from 547 in 2002 to 6625 in 2014 [22]. Among people seeking substance use disorder treatment for heroin, the proportion of patients reporting methamphetamine use increased nearly 500% between 2008 and 2017 [7].

Vital statistics records have also demonstrated dramatic increases in methamphetamine-related or involved deaths in the USA [23]. Methamphetamine-related deaths increased almost five-fold, from 0.8 per 100,000 in 2012 to 3.9 per 100,000 in 2018 [24]. Notably, a meaningful share of drug overdose deaths now includes combinations of opioids and stimulants and, particularly in the Western USA, opioids and methamphetamine are frequently consumed together [25, 26]. However, while opioid overdose has been clinically defined and death is understood to result from opioid-induced respiratory depression, methamphetamine-related fatalities are not as amenable to easy identification. Specifically, the toxicity of methamphetamine, levels of fatal blood concentrations, and the contribution of other substances and underlying comorbidities create a less straightforward picture when trying to understand methamphetamine-related deaths. A 7-year study of autopsies for methamphetamine-related deaths in Australia (2009–2015) found that in 83% of cases, other substances were detected in postmortem toxicology (mostly opioids and hypnosedatives); methamphetamine toxicity alone was identified as cause of death in a minority (6.1%) of cases [16]. Importantly, among the 1649 descendants in the Australian study, causes of death other than toxicity (e.g., coronary disease, cardiovascular injury, suicide, or injury) accounted for a large share of deaths. This suggests that effects other than acute methamphetamine toxicity from an overdose could explain many methamphetamine-related deaths.

Recent qualitative research has identified motivations for methamphetamine use and described the experiences of users [27–35]. However, less is known about the experiences of methamphetamine *overdose* among the current population of people using it. This knowledge gap has important implications for the implementation of effective public health strategies to reduce morbidity and mortality associated with methamphetamine use. Specifically, unlike naloxone administration for opioid overdoses, which is relatively straightforward and can be implemented by laypeople, including people who use drugs [36–38], there is no analogue to naloxone for the treatment of the acute effects of methamphetamine overdose. The presentation of a methamphetamine overdose is not as amenable to easy recognition and layperson response due to the complicated pathophysiology, role of polysubstance use, impact on multiple systems, and exacerbation of underlying chronic morbidity. This is particularly relevant as communities attempt to scale up harm reduction measures to reduce methamphetamine-related morbidity. Developing patient-centered methamphetamine overdose prevention strategies requires an understanding of how people who use methamphetamine define and experience the effects of methamphetamine overdose, and what they do to mitigate unwanted effects.

In the current study, we explore qualitative narrative accounts of methamphetamine overdose and strategies to reduce the undesirable effects associated with methamphetamine use. Data were drawn from a larger, mixed methods study to examine the patterns of methamphetamine use, perceived methamphetamine-related harms and benefits, and harm reduction strategies in two communities characterized by high rates of methamphetamine and opioid use [8, 39–45]. We conducted qualitative interviews with people who use methamphetamine to address the following research questions: (1) what are people’s experiences with methamphetamine use and overdose and (2) how do people who use methamphetamine manage the harms or undesirable symptoms associated with methamphetamine overdose?

## Methods

### Setting

This sequential mixed methods study was carried out in two Western states: Nevada and New Mexico, where media and local surveillance data indicate high rates of opioid overdose deaths and rising rates of methamphetamine-related death. Our university-based research teams in both locations collaborate with local community partners, including public health authorities, substance use disorder treatment providers, and harm reduction service agencies, through both formal and informal partnerships. For this study, community partners were included in the conceptualization, design, and implementation of the study and also allowed the research team to access to their facilities for participant recruitment and data collection activities. All study activities were approved by the University of Nevada, Reno (UNR) Institutional Review Board (IRB). The University of New Mexico IRB deferred oversight to UNR IRB under a single IRB agreement.

### Recruitment and data collection

Between December 2019 and February 2020, we recruited people utilizing a combination of street and agency-based outreach in both locations. Inclusion criteria were age 18 years and older and self-reported methamphetamine use in the past 3 months. Recruitment included providing flyers and conducting one-on-one outreach in locations known to be frequented by people who use methamphetamine, including encampments of people experiencing homelessness, syringe service programs, and bars. We also conducted chain-referral recruitment through existing participants.

Data were collected by trained qualitative interview staff using a loosely structured interview guide, in a private or semi-private location that was acceptable to the participants. In some cases, interviews were conducted in private rooms at our partner agencies’ offices, while in others they were conducted outside (e.g., in an encampment, at a participants’ dwelling). All participants completed the interview in English, though everyone was provided the option to have data collected in English or Spanish. Written informed consent was obtained from all enrollees. To guard against perceptions of coercion, participants were compensated $40 immediately upon providing consent and were reminded that they could end the interview at any time without question or consequences.

First, we collected a small set of quantitative demographic variables including age, gender, sex, ethnicity, race, residential location, homelessness, education, employment, recent incarceration, and access to healthcare services. Then, the qualitative interview began with broad questions about the respondents’ drug use, including reasons for using methamphetamine, current drug use patterns, and changes over time. Most relevant to the current analysis, we asked four questions related to methamphetamine overdose and other negative experiences: (1) What kinds of drawbacks or negative experiences are you having from your methamphetamine use right now, or have you experienced in the past?; (2) Have you or anyone you know experienced what you believe to be a methamphetamine overdose? What did that look like or feel like?; (3) Can you tell me about your experiences seeking medical care for any issues associated with your drug use?; and (4) What strategies have you used to reduce any harms or negative experiences associated with your methamphetamine use?

### Analysis

Interviews were digitally recorded and transcribed verbatim for analysis. After conducting quality assurance review and redaction of the transcripts, data were analyzed using an inductive thematic approach. Guided by our research questions, a single analyst reviewed all the transcripts and began by making a series of memos documenting initial impressions. Those memos were discussed with the entire research team, which includes researchers and service providers, some of whom have lived and living experience of substance use. The analyst developed a set of thematic codes, arranged into a hierarchical categorization scheme, which they applied systematically to all the transcripts. After an initial round of coding, the coded transcripts were reviewed and discussed with team members and codes were further defined and refined, while memos were expanded to capture emergent ideas. Finally, the analyst and one study PI, a mixed methods researcher with 20 years of qualitative research experience, collaboratively organized the output from the coding and identified commonalities across the narratives and illustrative quotes. Quotes are provided using a unique respondent identifier (e.g., “R13”), ethnicity, race, sex, age, location of interview, and drugs used most often by the respondent.

## Results

We interviewed 21 people (11 from Nevada, 10 from New Mexico). Respondents were 48% female and 52% male. In terms of race and ethnicity, 2 participants were black, 10 were white, 3 were multiracial, and 6 were Latinx. Median age was 35 years (IQR: 30–43). Just under half (48%) reported being homeless, 81% had completed 12 years or more of formal education, and 38% were employed full or part time. The majority (17/21) reported using mostly methamphetamine and opioids (including heroin, methadone, and prescription opioids); only four reported using methamphetamine alone.

### Experiences of overdose

When asked whether they or anyone they know had experienced what they believe to be a methamphetamine overdose, reports of fatal acute methamphetamine overdose were rare and some respondents asserted that a methamphetamine overdose is “not possible.” For example, when asked whether he had ever experienced a methamphetamine overdose, one respondent said:

“You can’t OD on meth. I don’t care what they say. You fucking nod the fuck out. Maybe you have a weak heart or something or it gives out, okay, then yeah, you die, whatever. But you should[n’t] be doing that [dying of an overdose].”

R13, Non-Hispanic/Latino, White, Male, 30s, Nevada, MA+heroin

He went on to distinguish the experience of “overdosing” from the experience of “overamping,” which he describes as the body “shutting down” due to the over-stimulation:

“They nod out. It’s called overamping. Overamping, okay? You hit, your body [inaudible] because you had so much energy, your body can’t take it. Your body will just shut the fuck down. Your heart cannot take that physical fucking rush and people will shut down, they go to sleep.”

R13, Non-Hispanic/Latino, White, Male, Nevada, MA+heroin

In only two of the 21 interviews did respondents describe what appeared to be a fatal methamphetamine overdose, and both accounts were told second hand (i.e., not directly observed by the respondent); in addition, they both seemed to describe the same event. Details about the actual event were sparse: the overdose victim was left alone, and the specifics of the death were unclear. In the remaining 19 interviews, respondents described psychological or physical effects of taking too much methamphetamine, but in general these were described as non-life-threatening experiences. In the sections that follow, we report in more detail the spectrum of symptoms associated with what many respondents described as “overamping.” We categorize these experiences in terms of whether they are experienced primarily as psychological or physical symptoms (though often there is overlap), and analyze each in terms of their desirability, uncomfortableness, level of concern about health/safety, and perceived need to seek emergency medical intervention.

### Psychiatric effects

One common set of experiences were psychiatric effects. For the most part, these effects were experienced along a continuum, from symptoms that participants described as less concerning such as memory lapses and anxiety to more worrisome symptoms including paranoia, delusions, and hallucinations. Respondents who had memory lapses explained that they were similar to alcohol-induced blackouts—they forgot what happened, but the experience did not put them in immediate physical jeopardy. One respondent described this experience by saying that four or five days had passed and they hadn’t realized that any time had passed. Another respondent told a story about purchasing a new car, experiencing a memory lapse, and leaving the car abandoned for several days. Three days later she recovered but could not recall where she left her new car. In cases of memory lapses, the respondents describe the experience having a rapid onset, like a switch clicked off. They might recall having a conversation up to a certain point and then forgetting how the conversation continued or what happened after that. They might be partying in one part of town and find themselves across town a day or two later. None of the respondents talked about experiencing falls or injury during these memory lapses, but one respondent did identify the vulnerability of someone in this state, saying:

“…but when you overamp and like that, it can be a scary thing because that’s when girls get raped or guys rape, whatever. You don't think about the consequences anymore. You’re so high that whatever matters in that moment is [inaudible] in that moment. That's it.”

R10, Non-Hispanic/Latino, White, Male, 20s, Nevada, MA+heroin

Our interview guide did not systematically ask participants about alcohol consumption. However, of the four respondents who mentioned memory lapses, only two said they consumed alcohol and one of them said they had cut back on alcohol use because it interfered with the effects of other drugs.

More concerning psychiatric effects included paranoia, delusions, and hallucinations. These sometimes happened independently, but could also occur concurrently (delusions/paranoia, delusions/hallucination, etc.). In many cases, respondents interpreted these effects as signs they needed to sleep, but not as life-threatening events that required medical attention. In the following passage a respondent describes an experience of paranoia that was on the less-worrisome end of the spectrum for him:

“That’s the level, like there’s a level of fear that comes from, for me anyway, I know some people turn into aggression, I turn into sort of introversion where I’m just super scared of everybody. I walk in the store, and oh, my god. Fucking everybody is looking, you son of a bitch.”

R14, Hispanic/Latino, White, Male, 30s, Nevada, MA+heroin

Later, this same respondent described a more worrisome experience in which he received medical care following a hallucination in which he believed he had killed someone in a car accident. The hallucinations and delusions started in a hotel room. In an attempt to wake himself: he broke things, went to the front office naked, and tried to pour water on himself. After calming him down a bit, the motel staff called 911 and he was transported to the hospital, where he continued to experience auditory hallucinations of law enforcement and ER staff conspiring to kill him.

When people sought medical intervention (usually from an emergency room or by calling 911, but sometimes also from service providers in the community), they experienced that little could be done other than to ride it out in a safe environment:

“We had went over there, took my sister over there [to the community program]. She was flipping out. They didn’t know what to do. They said, we don’t know what to do about the meth yet. It’s a big thing. No one knows how to address it. Everybody’s going on these trips and we don’t know how to reverse the trip.”

R4, Hispanic/Latina, Black, Female, 20s, New Mexico, MA+methadone

Other respondents described their hallucinations in spiritual terms. Some respondents saw some hallucinations as welcomed spirits, engaging with them as if methamphetamine had opened a new spiritual plane.

“I liked it. I liked it. It opened my mind to a lot of shit that I didn't see… I was talking to this other guy and he says that he sees like -- I don't know how to explain it -- like demons. He sees like this shit that you never would see on straight. And I've talked to more than one person. I don't know what it is. I don't know if it's something that fucks within your mind or if this shit is really there or what. I mean it’s just weird because a lot of people are saying the same shit. And I don't know if it just -- I don't know what it is. I was just curious. I always wanted to ask somebody because they say that when you do meth, it opens you to a level, whatever, like million people that can see shit. I don't know if that's true.”

R2, Hispanic/Latino, Black, Male, 20s, New Mexico, MA+heroin

Others talked about their hallucinations as more frightening, including one respondent who thought the devil was coming for her soul.

“Me and him started sliding and we went on a trip, both of us but I took a little bit harder and I started thinking that my husband was with another girl. I started seeing this other girl. Really, in reality, which there was no other girl. I started accusing … him of [cheating with] her. I started hearing noises. I would see it. I was seeing things, awful things like the devil and stuff was trying to attack me. It was awful. I’ve been on an awful trip. It was very scary, and it was awful, awful.”

R7, Hispanic/Latina, White, Female, 40s, New Mexico, MA+methadone

In the quotation that follows, the same man who described his own hallucinations in a positive and spiritual way, described his female partner’s hallucinations in a more negative way:

“But last night, for whatever reason, she just flipped out. And I couldn’t handle it no more. I told her, ‘You’re going to have to leave.’ It’s sad that that was in the middle of the night. It’s cold. But I couldn’t do it no more. I mean I wouldn’t even [put up with it with] my ex-wife and I just can’t. I can’t handle it. It’s sad. I feel bad for her because she might be pregnant with my child.”

R2, Hispanic/Latino, Black, Male, 20s, New Mexico, MA+heroin

Though we did not systematically ask participants how their route of administration impacted their experiences with methamphetamine overamping, nearly half of our sample identified injecting (compared to other routes of administration) as increasing the chance of delusions, and hallucinations. One person retells the story of his only time injecting methamphetamine. Later, he says this would be the last time he ever injected methamphetamine:

“When was the first day? Oh, I remember one time I shot it up. I felt like there was a worm in my eye. And I started flipping. I freaked out. I called the ambulance and I had them go pick me up. They looked in my eye. They did an ultrasound on the top of my eyelid and there was nothing in there.”

R3, Hispanic/Latino, White, Male, 40s, New Mexico, MA+heroin

As a result, some people stopped injecting and switched to smoking as a harm reduction response to their negative experiences of hallucinations:

“What’s amazing, to be honest with you, after I went on that trip, now that I smoke, it’s a whole different thing. I don’t get high like that no more. … Now, I’m normal. It just wakes me up a little. That’s it. I don’t trip. Thank, God. I don’t nothing. I’m normal, awake more.”

R7, Hispanic/Latina, White, Female, 40s, New Mexico, MA+methadone

### Physical effects

Reported physical effects included: a strong desire to sleep, cardiovascular symptoms, and uncontrollable movements of one’s face and extremities (which most respondents identified as “flailing”). Reflecting the account discussed earlier in which a male respondent said that overdose is simply the body “shutting down,” one of the less-concerning physical experiences was described as experiencing a strong desire to sleep:

“Basically, where you smoke yourself to sleep or you do too much in a shot and you just go to sleep… And you wake up. But when you wake up, you're energized. It's not like the heroin overdose is like where you feel shitty afterwards. You feel great afterwards. You don't feel like you’ve just been put through it. It just basically overamps you to where you turn off.”

R9 Non-Hispanic/Latina, White, Female, 30s, Nevada, MA+heroin

While this respondent described waking up feeling great, others reported feeling hungover or in withdrawal the following morning. Respondents did not describe experiences consistent with a vasovagal syncope (fainting); rather, they just experienced an uncontrollable desire to sleep. One respondent described it as sensory overload; after days of methamphetamine use and no rest, their body just could not handle further input and shut-down, like a computer restarting:

“Like I said, it overwhelms your senses and then your body. Your body just -- it’s going too fast. Your mind is going too fast or something. It shuts off, I guess. I don’t really know the actual reason why, but you'll see people starting to nod out when they’re doing it."

R10, Non-Hispanic/Latino, White, Male, 20s, Nevada, MA+heroin

In terms of cardiovascular effects, one respondent described feeling that his heart would burst through his chest. These experiences were described as alarming in the moment, but not life threatening. Most believed that these experiences would pass, and in fact, they did. Though rare, one respondent described offering street remedies for people experiencing cardiovascular effects:

“I got high with a couple of people that aren’t used to getting high the way we do. They’ll say, ‘Well, what…’ They’ll ask questions like, ‘What do you do if you get smoke too much? Or how do you know you’re having a heart attack?’ My advice to them was get a Benadryl and let it dissolve under your tongue and it does help. I’ve seen it help or glass of a milk. That calms the heart rate. I’ve seen people just freak out because they think they’ve done too much. Thankfully, I calm down by doing that. But other, if they don’t know what to do in a situation like that, then they probably have a panic attack and end up in the hospital.”

R8, Hispanic/Latina, White, Female, 30 s, New Mexico, MA + heroin.

Flailing, or as one respondent called it “hick-a-booing,” describes muscle spasms causing a person’s arms, legs, or jaw to move quickly, wildly, and without purpose or intent. In most interviews, respondents described “flailing” as an unpleasant experience.

“It’s when you do a shot and you’re coming really fucking high pretty much. It never goes away. You never stop acting that way. There was one time where I got like – I felt like I was [inaudible]. I can’t stop rocking back and forth. I can see myself doing it, watch myself doing it but I couldn’t stopped doing. I don’t know what it was. I just couldn’t fucking stop. Honestly, it was fucking – it scared the shit out of me…Like, “Stop doing that. Stop doing it.” But I can’t fucking – it was a psychosis thing. I started freaking out. Then that can get even worse when you do it more.”

R10, Non-Hispanic/Latino, White, Male, 20s, Nevada, MA+heroin

While unpleasant, few individuals expressed concern for their health or safety in a moment of flailing, most stating that they just needed to ride it out. For some of our respondents, the lack of control over their movements was itself a cause for concern, in that it signaled to others that the person was using methamphetamine. In some cases, flailing co-occurred with psychological effects, such as memory blackouts and delusions as described above.

### The role of sleep deprivation, dehydration, and hunger

Rather than attributing the psychiatric and physical symptoms to methamphetamine use or overdose, most respondents associated these symptoms with the cumulative effect of being awake for several days while binging on methamphetamine, during which time they became dehydrated or undernourished.

“Hydration. [crosstalk] Sort of a rationalization of sort of acceptance about okay, if the cops are behind me, they’re behind me. If they say it’s going [inaudible] and it’s just going to happen. I know it’s not actually going through but it was just saying okay, trying to pretend that you’re [inaudible], I guess. I mean literally staying hydrated and having nutrition [inaudible] I guess my body was just in a point of severe, severe like two, three days without water walking around”

R14, Hispanic/Latino, White, Male, 30s, Nevada, MA+heroin

This experience was described as unique to methamphetamine, and linked to the common experience of staying awake for several days while using methamphetamine. For example, one respondent compared the experiences using methamphetamine and cocaine:

“That paranoia comes with the meth after a couple of days awake. Of course, your mind's in a [inaudible] with you. You haven't slept. So that’s when paranoia comes in with that. Then for coke, it's just boom, hits you like that. Once you take that first hit, you get paranoia. You see a shadow. You fucking think someone's coming or they're out to get you or just whatever. It’s weird."

R8, Hispanic/Latina, White, Female, 20s, New Mexico, MA+heroin

In most cases, respondents did not experience symptoms until they had been awake for two to three days. For some, the onset of such symptoms signaled the need to get some rest and stop using for a while.

“After one or two days, I have to take a break because I just don’t like going on days without sleep. It’s just the mental side effects for me personally are just outrageous if not careful with it. I end up losing jobs because if you spooked out to go to work and I feel like just grandiose far out paranoia usually if I’m not careful with it.”

R11, Non-Hispanic/Latino, Black, Male, 20s, Nevada, MA+heroin

“Yeah. If I’m up for three days or whatever and I start looking at the door handle like that, I’ll think it starts to move, like someone’s going to come or starting the door lock going like, “Ta-da.” It’s crazy. Then nothing happens. Like I said, my friend’s been like, “Look, look.” She’s showing me something on her finger. You stay looking long enough and you start thinking you see it moving like little worm or whatever. Oh, my God. Then it’s like, “No, it’s not there. Look at that shit when you’re sober.”

R8, Hispanic/Latina, White, Female, 20s, New Mexico, MA+heroin

## Discussion

We interviewed 21 people who use methamphetamine in Nevada and New Mexico to elucidate experiences they described as methamphetamine “overdose” and the ways in which people manage or reduce their risk of experiencing negative effects. Our goal was to elicit PWUDs’ perspectives on this issue, which should be centered when informing funding, policy, and programmatic initiatives. Notably, nearly all of our respondents (17/21) reported use of opioids and methamphetamine—only 4 reported only using methamphetamine. When asked about their experiences of methamphetamine overdose, our respondents asserted that experiences of acute fatal methamphetamine-only overdose are rare and none described what could be interpreted as a polydrug overdose with methamphetamine and opioids.

It may be that the experience of methamphetamine “overdose” cannot be described using the same framework that is currently used to describe opioid overdose. The primary effects of opioid agonists such as heroin include analgesia, euphoria, and respiratory depression [46]. On the other hand, methamphetamine has both acute and chronic effects on the cardiovascular and cerebrovascular systems [20], and has been implicated in various underlying cardiac and cerebrovascular diseases that result in death [47]. While fatal opioid overdose manifests in the form of acute respiratory depression resulting quickly in death without intervention, methamphetamine “overdose” might be more accurately described as a constellation of psychological and physical symptoms that are experienced on a continuum from less to more severe. A more pragmatic and patient-centered approach might be to acknowledge and address the cumulative effects of methamphetamine use as a risk factor for the cardiovascular and cerebrovascular systems [48], while also addressing the positive and negative behavioral, physical, and psychiatric consequences of its use.

Of note, many of the symptoms described by our sample could be attributable to other causes, such as extreme sleep deprivation and/or polysubstance use, rather than the acute effects of methamphetamine use alone. Auditory and visual delusions and hallucinations have been experienced by individuals suffering extreme sleep deprivation [49], and lack of sleep is a risk factor for anxiety, and major depression [50, 51]. Therefore, it is difficult to determine if methamphetamine use itself, or the sleep deprivation brought on by its use, is the more proximal cause of symptoms experienced in our sample. Future research should determine if behavioral health interventions to improve sleep patterns could result in fewer negative psychological symptoms experienced by people using methamphetamine. Similarly, the memory lapses described by our participants as “blackouts” might be the result of excessive alcohol consumption. However, our data do not suggest that alcohol consumption was a major factor among those who described this experience.

A primary finding from our study is that, while accounts of acute fatal methamphetamine overdose were rare, respondents did experience a range of undesirable and potentially harmful symptoms, and respondents were aware of few clinical or harm reduction strategies to reduce those negative consequences. One respondent recommended the use of antihistamines for reducing cardiovascular symptoms associated with overconsumption, though clinical guidance for this pharmaceutical intervention is lacking. Otherwise, respondents identified general self-care strategies such as sleeping and staying nourished and hydrated. Some respondents were unable to identify any clinical or harm reduction therapeutics, a deficit which was also described when respondents sought medical care and found little help available. There is a critical need for efforts that collaborate with people who use methamphetamine to identify effective clinical and harm reduction strategies that draw upon and center their expertise, while preserving autonomy and self-determination [52, 53].

Our findings must be interpreted in light of a significant increase in polydrug use and polysubstance-related deaths documented in national surveillance data [7, 9, 23, 25, 26]. As noted previously, nearly all of our respondents also used some form of opioids, though their descriptions of methamphetamine overdoses did not appear to be related to opioids. Because many methamphetamine-related *deaths* may be more accurately attributed to polysubstance use (including combination of methamphetamine and opioids), structural intervention strategies that have been deployed to address the opioid overdose death crisis could be extended to create safer conditions for methamphetamine consumption. For example, overdose prevention sites (aka supervised drug consumption/injection sites) have effectively decreased opioid overdose mortality and other health consequences of injecting drug use in countries outside the USA [54–57]. Future research efforts should consider how these sites may benefit people who use non-opioid substances (alone or in combination with opioids) including methamphetamines and psychostimulants, both in terms of reducing mortality in the event of an overdose, and in terms of providing support and care for people experiencing unpleasant psychiatric or physical symptoms.

Drug testing programs and safe supply initiatives are other structural interventions that may possibly create safer conditions for methamphetamine consumption. Drug testing programs include models such as distribution of fentanyl test strips to PWUDs who use opioids, or onsite testing services using either rapid testing devices or mass spectrometry. Drug testing is feasible and acceptable, and allows PWUDs to test their own drugs and make informed decisions prior to using [58, 59]. However, fentanyl test strips can provide false positives when testing methamphetamine and in the presence of some adulterants [60], limiting their utility for PWUDs who use primarily methamphetamine or combine methamphetamine with opioids. Despite widespread concern about fentanyl-contaminated methamphetamine, the prevalence of fentanyl-positive methamphetamine samples tested with more accurate methods appears to be low. Data from the National Forensic Laboratory Information System reflects only 0.1% (*n* = 272) of methamphetamine seized in the USA during 2016 contained fentanyl [61]. One study of samples collected between 2017 and 2018 at a supervised drug consumption site in Vancouver, BC, found that 5.9% (*n* = 15) of methamphetamine samples contained fentanyl, using a FTRI spectrometer [62]. This highlights the need for accurate testing devices that can be used in real time by people who use methamphetamine. Some research suggests that drug checking programs may be less effective for the most vulnerable or marginalized PWUDs who cannot afford to replace tainted supplies [63–67]. Therefore, safer supply initiatives, which provide substances of known quality and dosage, should be evaluated for their effectiveness at reducing methamphetamine-related harms.

Another challenge related to the current wave of methamphetamine-related deaths is that medications to treat methamphetamine use disorder (especially among people with opioid use disorder) are still under development in the clinical trial pipeline [68]. Behavioral interventions such as contingency management and cognitive behavioral therapy show some promise but concerns about their durability and sustainability remain [69–74]. Therefore, there is an urgent need for expanded options for those desiring treatment for methamphetamine use disorder. Efforts to extend this knowledge base should be prioritized in the short-term.

Our findings should be interpreted in terms of some limitations. We employed a convenience sampling approach that included street- and agency-based outreach in communities with high volumes of people experiencing homelessness. While our sample showed diversity on important structural determinants of health such as housing and employment, our findings may not generalize to other communities or methamphetamine-using populations that are less accessible through these sampling methods, such as people with more stable housing. Our sample was also drawn from two Western US states with high rates of methamphetamine and opioid overdose-related deaths, and represent a combination of rural, urban, and urban-serving communities. However, important cultural and structural differences may limit the generalizability of our findings beyond these communities. Because our qualitative interviews sought to understand participants’ patterns of substance use (including polysubstance use and changes over time) and their personal experiences of and understanding about overdose, our findings should not be used to draw conclusions about the particular routes of administration, substances used, or combinations thereof that elevate or reduce risk of overdose. Importantly, while we did ask respondents specifically about witnessing fatal methamphetamine overdoses, few were described, and our conclusions are largely limited to non-fatal events.

## Conclusion

The USA is in the midst of a “fourth wave” of overdose deaths, increasingly attributed to methamphetamine used alone or in combination with opioids. When asked directly about their experiences of methamphetamine overdose, our respondents claimed that acute, fatal overdose is rare or even impossible. However, they did describe a number of undesirable symptoms associated with overconsumption of methamphetamine and had few clinical or harm reduction strategies at their disposal to reduce those undesirable effects. Addressing this current wave of drug-related deaths will require attention to the multi-level factors that structure experiences of methamphetamine “overdose,” and a collaborative effort with PWUDs to devise effective harm reduction and treatment strategies.

## Data Availability

Because of the sensitive nature of the information contained in the transcripts (e.g., details about illegal behavior) and potential for severe ethical, legal, and social consequences resulting from broken confidentiality, full transcripts will not be made publicly available. Redacted excerpts of the qualitative transcripts used in the current analysis will be made available to qualified researchers subject to review and approval by the appropriate Institutional Review Board(s). Requests can be made to the University of Nevada, Reno Research Integrity Office by calling + 1-775-327-2368.
